# Surface Response Methodology-Based Mixture Design to Study the Influence of Polyol Blend Composition on Polyurethanes’ Properties

**DOI:** 10.3390/molecules23081942

**Published:** 2018-08-03

**Authors:** Said Arévalo-Alquichire, Maria Morales-Gonzalez, Luis E. Diaz, Manuel F. Valero

**Affiliations:** 1 Energy, Materials and Environment Group, Faculty of Engineering, Universidad de La Sabana, Chia 140013, Colombia; saidaral@unisabana.edu.co (S.A.-A.); mariamorgon@unisabana.edu.co (M.M.-G.); 2 Doctoral Program of Biosciences, Universidad de La Sabana, Chia 140013, Colombia; 3 Bioprospecting Research Group, Faculty of Engineering, Universidad de La Sabana, Chia 140013, Colombia; luisdb@unisabana.edu.co

**Keywords:** polyurethane, polyol, mixture design, design of experiment, structure-properties relationship

## Abstract

Polyurethanes are materials with a strong structure-property relationship. The goal of this research was to study the effect of a polyol blend composition of polyurethanes on its properties using a mixture design and setting mathematic models for each property. Water absorption, hydrolytic degradation, contact angle, tensile strength hardness and modulus were studied. Additionally, thermal stability was studied by thermogravimetric analysis. Area under the curve was used to evaluate the effect of polyol blend composition on thermal stability and kinetics of water absorption and hydrolytic degradation. Least squares were used to calculate the regression coefficients. Models for the properties were significant, and lack of fit was not (*p* < 0.05). Fit statistics suggest both good fitting and prediction. Water absorption, hydrolytic degradation and contact angle were mediated by the hydrophilic nature of the polyols. Tensile strength, modulus and hardness could be regulated by the PE content and the characteristics of polyols. Regression of DTG curves from thermal analysis showed improvement of thermal stability with the increase of PCL and PE. An ANOVA test of the model terms demonstrated that three component influences on bulk properties like water absorption, hydrolytic degradation, hardness, tensile strength and modulus. The PEG*PCL interaction influences on the contact angle, which is a surface property. Mixture design application allowed for an understanding of the structure-property relationship through mathematic models.

## 1. Introduction

Polyurethanes (PUs) are a special group of polymers with a wide range of applications in industry, including adhesives, aircraft, furniture, insulation, construction and biomedical applications. Their versatility is explained by the variety of properties expressed by PUs, which are closely related with their composition [1]. The primary synthesis begins with the reaction between a polyhydroxyl donor called a polyol and an isocyanate to form a urethane bond. The resulting structure depends on the ratios of the compounds. PUs can be obtained in flexible and rigid foams, thermoplastics, coatings, adhesives, sealants, elastomers, waterborne dispersions [2] and hydrogels [3]. The three main components in polyurethane synthesis are the polyol, isocyanate and chain extenders or crosslinkers. Polyols form the soft segment, and isocyanate and a chain extender or crosslinker form the hard segment. The nature of the polyol and the hard segment content in PUs can regulate both their bulk and surface properties [1]. 

Studies of polyol composition have been carried out. Trzebiatowska et al. obtained polyurethanes from the glycerolysate of recycled polyurethanes and poly(ethylene-butylene) adipate diol. As the content of the recycled component increased, the swelling ratio decreased, and the crosslink density simultaneously increased, resulting in the rise of glass transition and storage modulus at room temperature. Thermomechanical stability, tensile strength, elastic modulus and hardness of the PUs also increased and elongation at break decreased with the incorporation of glycerolysate. The original polyol used was tri-functional, and as such, it is possible to have more linkages per polyol molecule. Additionally, more networks could be created in this material as its structure is more branched. These properties can result in a reduction in polymer chains movement [2].

Bil et al. worked with aliphatic poly(ester-urethanes) from poly(ε-caprolactone) diol having different molecular masses (*M* = ~530, 1250 and 2000 Da), cycloaliphatic diisocyanate 4,4′-methylenebis (cyclohexyl isocyanate) and ethylene glycol as a chain extender. Changes in the macromolecule order, with increasing hard segment content, were observed via modulated differential scanning calorimetry. Depending on the hard segment content, gradual variations in the polyurethane surface properties were observed. Furthermore, as the content of the hard segments increased, the polyurethane surface exhibited more phase separation, a higher content of urethane moieties and higher hydrophilicity [4]. Moreover, previous work in our lab has been focused on the effect of polyol as well. Uscategui et al. evaluated the effects of the type of polyols, derived from castor oil by transesterification with pentaerythritol, with the incorporation of low concentrations of chitosan on the mechanical and biological properties of the polymer. The goal of this study was to obtain suitable materials in the design of biomaterials showing that increasing physical crosslinking increased the mechanical and adhesive properties, and bacterial inhibition depended on the polyol and percentage of chitosan [5]. 

Additionally, chain extenders and crosslinkers could tune the mechanical properties and hydrolytic stability of PUs. They are low molecular weight compounds that produce elastomeric behavior in PUs. Difunctional molecules are considered chain extenders, and higher functionalities are classified as crosslinkers. Shoaib et al. studied the effect of amino acid incorporation as chain extenders on the biocompatible and biodegradable properties, and pH responsive drug delivery of polyethylene glycol-based PUs. PUs showed swelling ability in the range of 4–13% indicating amorphous nature and reduction of intermolecular density, enhanced of hydrolytic degradation (20–38%) due to hydrolysable bonds like ester bonds, and high level of cell viability (>90%). Additionally, arginine had lower values of release at pH 7.4 as a chain extender, which resulted in less toxicity while fulfilling the requirements. However, at lower pH, behavior change due to the presence of free amino groups in arginine, which were protonated in the presence of acidic conditions, resulted in greater swelling and solvent penetration, thus increasing drug release [6]. 

Most of those works reported valuable knowledge about the effects of polyols and chain extenders. However, most qualitatively described the interactions between the components of the PUs based on the performance of some properties or, in some cases, just analyzed the behavior of one parameter. To establish the impact of these interactions quantitatively, design of experiment can be used. Design of experiment is a well stablished concept for the planning and execution of informative experiments [7]. Experimental design is a specific set of experiments defined by a matrix composed by the different level combinations of the variables studied [8]. One type of design applications concerns the preparation and modification of mixtures. This involves the use of mixture designs to explore the effect on mixture properties [7]. Experimental design has advantages such as cost and time economy, a reduced number of runs required to analyze the effects and the analysis of more than one factor at a time [9]. In the polymer field, Olivato et al. proposed a mixture model to evaluate the effect of tartaric acid on the properties of starch/poly(butylene adipate co-terephthalate) blown films plasticized with glycerol. They found that the interaction between the polymer and tartaric acid has a positive effect on the tensile strength and puncture force. Additionally, a greater proportion of tartaric acid increased Young’s modulus and contributed to the reduction in water vapor permeability [10]. Abolghasemi Fakhri et al. studied ternary-based nanocomposites based on polystyrene, nanoclay and zinc oxide nanoparticles for food packaging material using a central composite design-based response surface methodology. They found a synergistic effect between nanoclay and zinc oxide, resulting in the improvement of the mechanical and color properties of the mixture [11]. 

Specifically, in the polyurethanes field, Li et al. examined bio-based flexible polyurethane foams using bio-polyol, extracted from the fast pyrolysis oil of wheat straw, foaming processes such as the bio-polyol to petroleum-based polyol ratio, the polymethylene polyphenylene isocyanate to polymeric diphenyl-methane diisocyanate ratio, and the crosslinking agent content was optimized using response surface methodology. Resilience was found to increase with an increasing amount of petroleum-based polyol, while for bio-polyol, resilience was decreased up to a medium dosage after which there was a slight increase. However, increasing the amount of polymethylene polyphenylene isocyanate along with the petroleum-based polyol had a positive influence on the resilience of PUs, though it declined at a high dosage of polymethylene polyphenylene isocyanate [12]. Zhao et al. fabricated expanded thermoplastic polyurethane bead foams with supercritical CO_2_ as a blowing agent. They studied the influences of saturation pressure, temperature and soaking time and their interactions on foaming behavior through a response surface methodology based on the Box-Behnken design. This showed that saturation temperature was the most significant parameter affecting the expansion ratio, shrinkage ratio, and cell morphology of PUs foams. There was an interaction effect between saturation temperature and soaking time, wherein the expansion ratio of the foams was more sensitive to changes in soaking time at higher temperatures. As the soaking time increased, the shrinkage of PUs foams increased first and then leveled off, and the cell diameter decreased significantly [13].

Despite interest in understanding the structure-property relationship of polyurethanes, to the best of our knowledge there are no reports regarding the application of design of experiments to evaluate the influence of monomer composition on properties of polyurethanes. Thus, the goal of this research is to evaluate the effect of polyol bled composition on the properties of polyurethanes, including water absorption, hydrolytic degradation, contact angle, tensile strength and modulus, through mixture design and data regression, generating mathematic models to better understand the structure-property relationship in PUs. 

## 2. Results & Discussion

### 2.1. Polyurethane Synthesis and Chemical Structure

In this work, effect of polyol composition and crosslinker concentration was evaluated on different properties like: thermal behavior, hydrophilicity and hydrolytic degradation and mechanical behavior based on tensile test and hardness. Polyethylene glycol (PEG) and polycaprolactone diol (PCL) were used as polyols whereas pentaerythritol (PE) as crosslinker. Different concentration of those were studied. From this point, polyol blend should be understood as the combination of PEG, PCL and PE. PUs were synthetized by two steps polymerization: first, each polyol blend, with the composition described by the mixture design, was mixed with the IPDI and allowed to react. Further, a solution of crosslinker was add. Finally, bubbles were removed and poured into a cylindrical glass mold.

**Figure 1:**
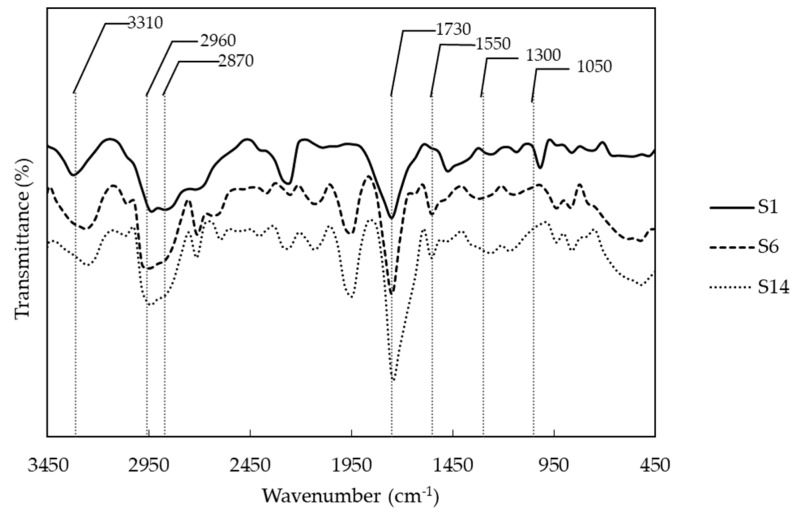
Representative infrared spectra of polyurethanes with different combination of polyols. (S1) PCL and PE, (S6) PCL, PEG and PE, and (S14) PEG and PE.

Successful of polyurethane synthesis was evaluated by FTIR (Fourier transform infrared spectroscopy). Figure 1 shows the functional groups of some synthetized polyurethanes with different polyol combinations. In general, polymers show the following peaks: at approximately 3310 cm^−1^, we observed the overtone related with the stretching of N-H bound from urethane and, near 1550 cm^−1^, the original signal. The asymmetric and symmetric vibration signals of -CH_2_ from the soft segments of the PCL and PEG and PE, from the hard segment, were observed at 2960 and 2870 cm^−1^. At 1730 cm^−1^, we can see the characteristic peak of urethane bound related with -C=O stretching. Additionally, between 1300 and 1050 cm^−1^, there are two peaks related to the asymmetric and symmetric stretching of -C-O-C- group, which is also part of the urethane bond. Finally, below 1050 cm^−1^, there are peaks related to the vibration of the aliphatic ring from the IPDI. According to the above, polyurethanes were obtained with an integration of polyols, crosslinker and isocyanate structures. Despite the successful synthesis of PUs, NCO groups vibration was observed around 2250 cm^−1^ in some PUs due to some NCO groups that didn’t react. 

### 2.2. Mixture Design

In this work, we applied a mixture design to study the influence of polyol blend composition on properties of polyurethanes. It had 9 design points, PUs with different polyol blend composition, and seven repetitions of some design points. Mixture design used different point types to evaluate the experimental space. This mixture design could be divided into three series: the first series (extreme of the experimental space) includes PUs with PCL as the major component named S1, S2, S3, S4, S5. The second lays in the middle of the experimental space (S6, S7, S8, S9, S10, S11). Finally, the opposite extreme where PEG was the major component (S12, S13, S14, S15, S16) was tested.

Reponses were analyzed by Box-Cox distribution. Transformations were carried out according with Box-Cox suggestions. Therefore, the inverse square root of water absorption and the square root of hydrolytic degradation and tensile strength were applied. Non-transformations were made on contact angle, modulus, hardness, kinetics of water absorption and hydrolytic degradation, and thermal responses. Additionally, DFFITS tests were applied to identify influential points. For contact angle, 2 influential points were deleted from the analysis (S15 and S16). The responses of S11 and S7 were removed for the kinetics of water absorption and thermal response, respectively Special cubic models were fit for each response for the regression models. Model regression based on the least square was used to compute the coefficients. The equations are presented in Table 1. Models were evaluated by ANOVA. The ANOVA test for the fitted models (see Table 2, Table 3 and Table 4) demonstrated that the model sum of squares was statistically significant at a 95% probability level (*p*-value < 0.05) for the properties studied. In addition, lack of fit was not significant at a 95% probability level (*p*-value > 0.05) in all cases. Both, the model sum of square and lack of fit indicated that the models were adequate. Likewise, fit statistics confirmed good fitting for all properties. According with Olivato et al. an R-square value higher than 0.7 suggests a good fit with the experimental data [10]. In this study, all R-square values were over 0.96. Additionally, the adjusted R-square value was calculated to be greater than 0.94, demonstrating that the variations in the responses can be explained by the relationships obtained. In the same way, the predicted R-square value determined how well the model predicts response. Here, the values were over 0.8. Therefore, the models obtained herein are promising for the evaluation of the structure-property relationship of polyurethanes.

### 2.3. Hydrophilicity and Hydrolytic Degradation 

**Figure 2:**
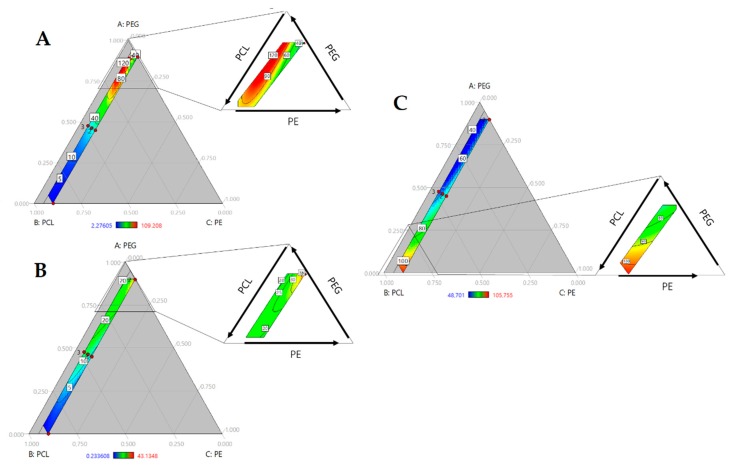
Ternary contour plots of (**A**) Water absorption (%); (**B**) Hydrolytic degradation (%); (**C**) Contact angle (°).

To evaluate the hydrophilicity, water absorption and water contact angle were evaluated. Figure 2A shows the contour plot of water absorption (non-transformed). There is an increased swelling with an increase in the PEG fraction. Conversely, swelling decreased with the amount of PCL. PEG and PCL are hydrophilic and hydrophobic polyols, respectively. These responses indicated that the nature of the polyols regulated the swelling response. Despite the urethane bonds, polyurethane chains conserve the structure of polyols, influencing the polyurethane response. Here, we observed how the incorporation of PEG increased the water absorption to levels that were approximately 30% due to the hydrophilic nature, and the largest amount of PCL showed the lowest swelling. 

Likewise, an increase in the PE fraction involved a reduction in swelling. PE added to the hard segment and increased the crosslinking of the chains, causing a reduction on chains mobility [2]. As observed in Figure 2A, where increasing of PE reduced the red area, or swelling, while a lower amount of PE showed a higher percentage of water absorption. According with Shoaib et al. (2018), a higher value of water absorption indicated an amorphous nature and decreased intermolecular density. In PUs, the hard segments acted as a twisting path for the diffusion of water molecules and the absorption increased with an increase in chain flexibility, providing more space for water molecules [6]. 

Checking the ANOVA results (see Table 2), the triple interaction (PEG*PCL*PE) was significant and confirmed by the standard regression coefficients, in which the triple interaction coefficient was the highest between the significant terms (β = 7.65). That is consistent with the analysis of the ternary plot (Figure 2A), concluding that the bulk composition of polyurethanes regulated the water absorption.

Contact angle measures the affinity between the solvent and material surface. Here, water was used as a solvent; therefore, wettability was evaluated. Lower angles indicate enhanced wettability. The surface is considered hydrophobic when contact angles are over 90° [14]. In Figure 2B, we can observe a decrease in the contact angle with an increase in the PEG content. The hydrophilic nature of PEG improved the interaction between water and PUs. Conversely, a rise in PCL increased the angle due to hydrophobic character of the polyol. PCL concentrations over 75% produced hydrophobic surfaces. However, this property did not change with the PE concentration. According to the ANOVA of regression model (Table 2), there is a significant effect for PEG*PCL interaction. This interaction had a negative impact on response, agreeing with the standard regression coefficients (β = −61.04) producing polymers with lower contact angle. Water contact angle is a surface property, and the model suggests that the soft segments of PUs regulate this property. 

Hydrolytic degradation occurred after swelling. Water enters the PU matrix. Chain scission takes place through hydrolysis, in which water molecules may facilitate the cleavage of some bonds such as urethane and esters [15]. As mentioned previously, this property is related to the swelling of water and hydrophilicity [16]. Thus, in Figure 2C, the largest amount of PCL had the lowest weight loss. Yet, increasing PEG content represents an increase in degradation. Hydrophilicity increased with contact angle, and a rise in water uptake resulted in an enhancement of degradation. However, there is a yellow to red area with the growth of PE at highest PEG concentrations. The addition of crosslinkers increased the urethane bond content, which has a hydrolysable ester bond. Furthermore, PEG is hydrolysable and has degradation products on its surface, making it highly hydrophilic and increasing the rate of degradation [6]. This suggests that swelling and hard segment content mediated degradation. As such, the lowest degradation rate occurred with higher concentrations of PCL, where the swelling is reduced. Model regression ANOVA showed that the triple effect (Table 2) was significant. It suggested that three components regulate degradation. As with water absorption, hydrolytic degradation is another bulk property that is directly regulated by its composition. 

**Figure 3:**
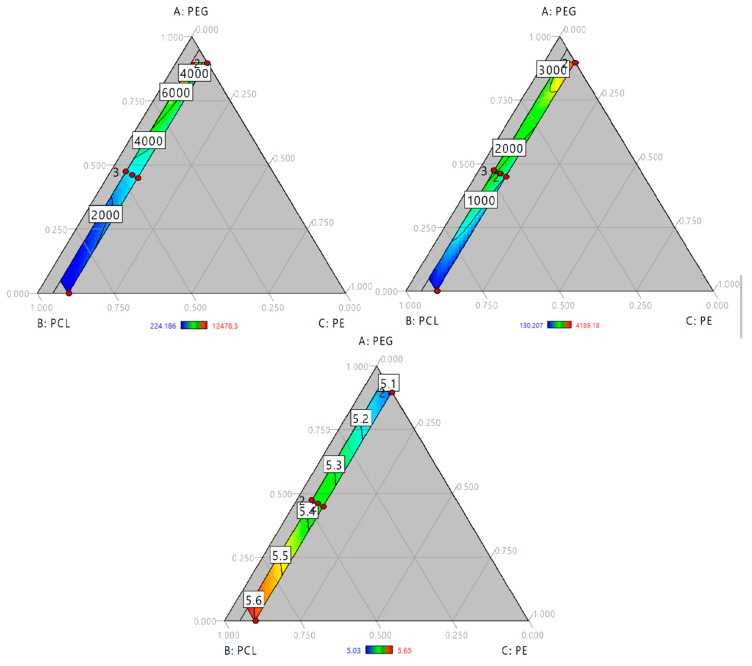
Ternary contour plot of area under the curve of (**A**) Water absorption kinetics (h); (**B**) hydrolytic degradation kinetics (h) and (**C**) First derivate of thermogravimetric analysis (1/°C).

Time vs. water absorption and hydrolytic degradation studies (data is presented in Tables S2 and S3) were carried out and area under the curve of each sample was calculated. The effect of polyol blend composition on the kinetics of water absorption and hydrolytic degradation was studied by a model regression. Our findings confirm the behavior described for water absorption and hydrolytic degradation for 48 h. In the first case (see Figure 3A) PUs with large swelling had the largest area under the curve. In the ternary plot there is a red area at greater values of PEG and lower of PE like Figure 2A. In the case of hydrolytic degradation described behavior like hydrolytic degradation at 48 h (See Figure 3B). Lowest values of area under the curve relates with lowest weight loss. ANOVA (Table 3) shows that triple interaction is significant for both responses. This triple interaction had a negative influence in water absorption kinetics and a positive effect on degradation kinetics.

Finally, measurement of area under the curve of those kinetics could be understood as the hydrolytic stability of PUs and. Higher values of area under of water absorption kinetics are related with largest values area under the curve of degradation kinetics indicating that PUs are affected by hydrolytic degradation.

### 2.4. Thermal Behavior

**Figure 4:**
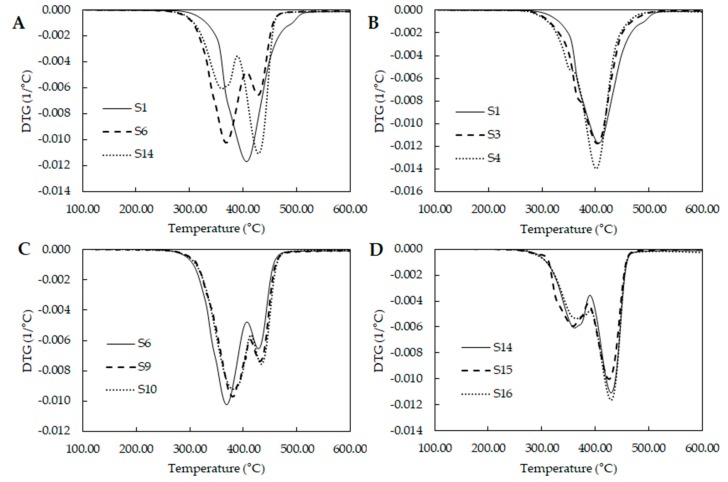
Derivate thermogravimetric curves for different polyol blend combinations (**A**) Comparison of DTGs from the extremes and middle design points; (**B**) DTGs of design points where PCL is the major component; (**C**) DTGs of middle design points and (**D**) DTGs of design points where PEG is the major component.

Thermal analysis was carried out by thermogravimetric analysis (TGA). Curves were normalized with respect to initial sample weight. First derivate was calculated for each sample. Figure 4 shows the thermal degradation of some PUs with different mixtures of polyols. According with Sui et al. polyurethanes degrade in three steps, first, the hard segments degradation between 250 to 320 °C, following by the two products of the cleavage of the urethanes linkages which are (i) the small molecular weight compounds linked to urethane bond like the crosslinker and (ii) the soft segment chains [17]. 

Figure 4A compares the behavior of samples from the extremes and middle of the experimental design. S1 seems to describe one degradation stage, while S6 and S14 shows two stages. S16 shows and increase on second stage compared with S6. Studying the curves from the extreme design points where PCL is the major component (Figure 4B), they present a shoulder around 350 °C. S1 have the largest amount of PE which produce PUs with large amount of hard segment. This could produce an overlapping of the three stages due to the large amount of PUs pyrolysis. S3 and S4 have lower levels of PE and they show better defined shoulders suggesting that the shoulder is the degradation of the hard segments. 

By other hand, Figure 4C shows the behavior of the samples from the middle of the experimental space. Here, two separate stages could be identified. First one associated to the hard segment degradation and the second over the 400 °C with the soft segment degradation. Figure 4D showed the behavior of the extreme points where PEG is the major component. The same two stages could be observed, however second stages had larger area under the curve than curves in Figure 4C, it could suggest that higher amount of hard segment was produced in the middle points than the points where PEG is the major component. Additionally, related the crosslinking density with the thermal stability and extreme points where PCL is the major component could present the highest content of hard segment and the highest thermal stability.

As for the onset temperatures (Table 5), PUs were thermally stable under 220 °C. Polymers with higher amount of PE showed higher values. Additionally, polymers with higher concentrations of PCL had higher temperatures. The addition of PEG reduced the onset temperatures. Król et al. described that polyesterurethanes are more stable thermally than polyetherurethanes; ester bonds undergo decomposition within 390 to 440 °C while ether decompose between 360–400 °C [18]. Our finding confirms that behavior, PCL is a polyester and the extreme points where PCL was the major component described the higher values of onset temperature.

To study the effect of composition, regression of onset temperatures was carried out. However, poor fitting properties were obtained (R-square = 0.64). So, the absolute normalized area under the curve (nAUC) of the DTG curves were fitted as approach to index the thermal stability in terms of polyol composition. Ternary plot (Figure 3C) showed a red area with the increase of PCL and PE. Addition of PEG to the mixture reduce the nAUC. This is accorded with the behavior of onset temperature suggesting that higher values of nAUC represents higher thermal stability and lower values represents lower thermal stability of polyurethanes. ANOVA (Table 3) showed a linear model where the terms of each component were significant. Standardized coefficients showed that the influence of composition follow PE > PCL > PEG.

### 2.5. Mechanical Properties

**Figure 5:**
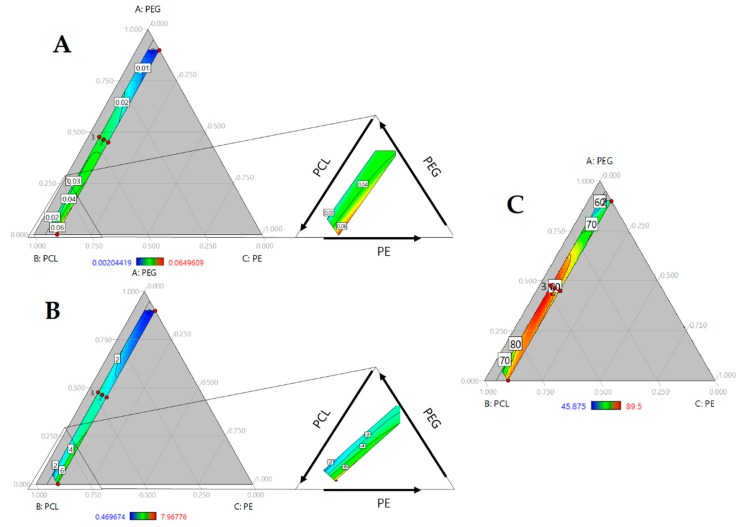
Ternary contour plots of (**A**) Modulus in MPa; (**B**) Tensile strength in MPa and (**C**) Shore A Hardness as function of PEG, PCL and PE mass fraction.

The mechanical performance of PUs can be regulated by hard segment content or polyol characteristics, such as molecular weight and hydroxyl index. In Figure 5A,B, modulus and tensile strength are presented. In both cases, the properties increase with the amount of PCL and PE. As mentioned previously, PE increases the hard segment content in PUs, modulating their mechanical response. The hard segment interacts through hydrogen bonds, generating crosslinking [19], and a reduction in chain movement could enhance mechanical performance. Increasing the PE concentration promoted urethane linkage formation. Additionally, PE functionality of 4 allowed for the formation of a branched structure. It can reticulate the structure and increase crosslinking. PE influence can be observed by the standard regression coefficient, β = 102.12 and β = 0.19 for tensile strength and modulus, respectively. 

Additionally, model regression ANOVA showed a significant triple interaction (Table 4), suggesting that both mechanical properties are mediated by all three components.

Hardness of the PUs was evaluated too. An increase of PE concentration results in a rise of hardness and largest values was reached at a high concentration of PCL (Figure 5C). ANOVA (see Table 4) showed significant triple interaction with a positive influence, according with standardized coefficients. Our results suggest that, in addition to crosslinking, the molecular weight of polyols mediated in mechanical properties.

Regression of elongation at break was carried out, but it showed low fitting properties (R-square = 0.35). This property was not analyzed in this work. 

### 2.6. Model Validation

Our models were validated by the check point method. Composition of check materials were selected because they were in the experimental space and were different than design points. The response of check materials in properties like water absorption, hydrolytic degradation, tensile strength and contact angle was carried out in Design Expert 10 based on the confidence intervals at 95%. The response of the materials adjusts to the model’s responses. It suggests that our models can predict the structure properties relationship of polyurethanes based on the composition of the polyol blend and can be used to navigate the design space and optimize desired properties. 

## 3. Materials and Methods 

### 3.1. Materials

The following reagents were used for PUs synthesis: polycaprolactone diol (PCL) with average molecular weight of Mn~2000, isophorone diisocyanate (IPDI), *N*,*N*-dimethylformamide (DMF) were purshased from Sigma-Aldrich (St. Louis, MO, USA). Polyethylene glycol (PEG, Mn~1000) was from Merck KGaA (Darmstadt, Germany), and pentaerythritol (PE) was from Alfa Aesar (Heysham, UK). For characterization analysis, phosphate buffer saline (PBS) was purshased from VWR (Radnor, PA, USA). 

### 3.2. Polyurethane Synthesis

Polyurethanes were synthetized via a two-step polymerization. PCL and PEG were used as polyols, PE and IPDI as crosslinkers and as a NCO source, respectively. Each weighed polyol sample was preheated at 110 °C and mixed with 10 mL of DMF, and then IPDI was added keeping the NCO/OH ratio of 1 and allowed to react for 15 min at 70 °C with agitation. In parallel, PE was diluted in 10 mL of DMF at 110 °C with agitation. Next, the PE solution was added to the prepolymer. A vacuum was applied to remove air. Finally, each polymer was poured in cylindrical molds of 100 mm of diameter and 2 to 3 mm of height and cured at 110 °C for 12 h. After the curing period, PUs were washed in a 50% (*v*/*v*) ethanol-water solution for 1 h to remove mold released and dried at 80 °C.

### 3.3. Design of Experiment

In this work, the effect of the polyol blend composition on several properties of polyurethanes was studied through mixture design. PCL, PEG and PE composition, in mass fraction, was varied in the ranges shown in Table 6.

A special cubic model was used to study linear, double and triple effects and the sum of composition must be one. Table 7 describes the polyol blend and type, as well as the composition of each material. Polyol bend refers about the compounds combination if there are two components in a blend, is a binary mixture or if all components are in the blend, it is classified as all components. Type refers to the location of each polyol blend in the experimental space, vertexes are the points were the limits of each components match, overall centroid is the point where the middle value of the limits ranges match and the edge centroids are the points where two components are in middle of the ranges and the last one is in one of the limits, each property studied was measured three times, and the average value was used for the model regression. Data analysis and model regression were carried out in Design Expert v10 (Stat-Ease, Inc., Minneapolis, MN, USA). Before regression, outliners and influential point were evaluated by Cook’s distance and DFFITS test. Box-Cox distribution was evaluated, and recommended transformation was applied. Model regression was made by minimum least squares. Analysis of variance (ANOVA) was carried out for model validation and the evaluation of significant terms. 

### 3.4. Polyurethane Characterization

The chemical structure of the PUs was evaluated by Fourier-transform infrared spectroscopy (FTIR) over a range of 400 cm^−1^ to 4000 cm^−1^ on a Nicolet iS10 instrument (Thermo Scientific, Waltham, MA, USA). The experiment was setup in 32 scans and resolution of 4. 

Thermal behavior was studied by thermogravimetric assay in a TGA/DSC 1 (Mettler Toledo, Columbus, OH, USA). The method consisted of two steps. The first was at 105 °C for 1 h to remove water and solvent traces. The second a dynamic heating from 105 to 600 °C at a rate of 10 °C/min. Tests were carried out under nitrogen atmosphere with a flow of 180 mL/min. Curve were normalized respect to initial weight of the sample. DTGs, onset temperatures and normalized area under de curve were calculated using the STARe software provided by the TGA/DSC1 supplier. 

Contact angle was determined using a MobileDrop (KRÜSS GmbH, Hamburg, Germany) with distilled water at room temperature and the sessile drop method. At least 3 measurements per polymer were taken, and the average of those was reported. 

Water absorption was evaluated by soaking each sample in triplicate in 1 mL of PBS 1× Samples were incubated for 48 h at 37 °C. Swelling was calculated with the initial weight (Wo) and the final weight (Ws), according with the following equation:

%Water absorption = (Ws − Wo)/Wo,(1)

Hydrolytic degradation was analyzed by soaking the samples in PBS 1x as before. After 48 h, samples were dried in an oven at 80 °C until a constant weight was reached. The dried weight was recorded (Wi), and degradation was calculated as follows:

%Degradation = (Wo − Wi)/Wo,(2)

Kinetics of water absorption and hydrolytic degradation were carried out, following the method described for each property. At least 5 time points were recorded in interval of 120 h. Curve were analyzed based on the %Water absorption and %Degradation. Under the curve area was calculated using the trapezoid method. 

The mechanical properties of PUs were assessed by uniaxial tensile test with EZ-XL universal test machine (Shimadzu, Kyoto, Japan) at 10 mm/min. Ultimate stress, modulus and elongation at break were recorded. Shore A hardness was measured using a shore A durometer. 10 measurements were recorded for each sample and the average was used for the model regression. 

## 4. Conclusions

Application of a mixture design allowed us to establish mathematic relationships for the effects of polyol blend composition on properties of polyurethane obtained from PEG and PCL (polyol), PE (crosslinker) and IPDI (isocyanate). An ANOVA test and fit statistics suggested a good fit and predictive potential with those equations. Water absorption, hydrolytic degradation and contact angle responses were modulated by the polar nature of polyols and crosslinker concentrations. However, tensile strength, modulus and hardness could be mediated by other characteristics of the polyols such as molecular weight and hydroxyl value, together with the crosslinker concentration. From a statistical perspective, a triple interaction (PEG*PCL*PE) with water absorption, hydrolytic degradation, tensile strength, hardness and modulus was significant, but not for contact angle, where the PEG*PCL interaction was significant. This suggests that bulk properties such as those in the first group are regulated by the three components in the composition, but contact angle, as a surface property, is mediated by the polyol nature. Thermal stability was evaluated by the area under the curve of the DTGs. It shows that increases of PCL and PE improved stability. Additionally, hydrolytic stability was evaluated by the area under the curve from the water absorption and hydrolytic degradation kinetics where similar behavior was observed, showing that the area under the curve could be used to identify the effects of variables. 

To conclude, this methodology allowed for a better understanding of the structure-property relationship through a mathematic model, identifying the main effects of the polyol blend composition on the final properties. These models look promising for optimization to design a polyurethane with target properties, and the mixture design could be extrapolated to other components such as isocyanate and the NCO/OH ratio.

## Figures and Tables

**Table 1:** Equations representing the models for the five properties studied.

Property	Units	Model Equation
Thermal Stability (Th)	1/°C	Th = 5.134 × PEG + 5.700 × PCL + 4.707 × PE
Water Absorption (WA)	%	1/sqrt(WA) = 0.047 × PEG + 0.534 × PCL + 7.872 × PE − 0.101 × PEG × PCL − 7.233 × PEG × PE − 6.928 × PCL × PE − 10.499 × PEG × PCL × PE
Contact Angle (CA)	°	CA = 2.09 × PEG + 110.073 × PCL + 220.13 × PE − 75.353 × PEG × PCL + 469.808 × PEG × PE − 182.699 × PCL × PE
Hydrolytic degradation (Deg)	%	sqrt(Deg) = −1.077 × PEG − 3.884 × PCL − 455.857 × PE + 23.734 × PEG × PCL + 585.13 × PEG × PE + 557.423 × PCL × PE − 278.709 × PEG × PCL × PE
Water Absorption kinetics (KWA)	h	KWA = 28,562.969 × PEG + 3753.102 × PCL + 530,986.252 × PE − 43,858.222 × PEG × PCL − 835,976.018 × PEG × PE − 624,450.94 × PCL × PE + 523,435.575 × PEG × PCL × PE
Hydrolytic degradation kinetics (Kdeg)	h	Kdeg = −525.686 × PEG − 2362.406 × PCL − 300,106.634 × PE + 14,011.812 × PEG × PCL + 383,372.797 × PEG × PE + 358,429.093 × PCL × PE − 197,168.276 × PEG × PCL × PE
Tensile strength (TS)	Mpa	sqrt(TS) = 0.899 × PEG − 0.123 × PCL + 77.941 × PE + 7.793 × PEG × PCL − 86.277 × PEG × PE − 55.399 × PCL × PE − 85.572 × PEG × PCL × PE
Modulus (E)	MPa	E = −0.012 × PEG − 0.043 × PCL − 0.912 × PE + 0.228 × PEG × PCL + 1.184 × PEG × PE + 2.145 × PCL × PE − 2.659 × PEG × PCL × PE
Hardness (HD)	Shore A	HD = −21.324 × PEG + 0.643 × PCL − 4231.919 × PE + 372.187 × PEG × PCL + 5662.460 × PEG × PE + 5682.430 × PCL × PE − 3878.500 × PEG × PCL × PE

**Table 2:** ANOVA test of model regression for water absorption, contact angle and hydrolytic degradation.

Source	Water Absorption						Contact Angle						Hydrolytic Degradation					
	SS	DF	MS	F	*p*-Value		SS	DF	MS	F	*p*-Value		SS	DF	MS	F	*p*-Value	
Model	6 × 10^−1^	6	1 × 10^−1^	3 × 10^2^	<0.0001	a	5 × 10^3^	5	1 × 10^3^	4 × 10^1^	2 × 10^−5^	a	6 × 10^1^	6	1 × 10^1^	1 × 10^2^	1 × 10^−7^	a
Linear Mixture	5 × 10^−1^	2	2 × 10^−1^	8 × 10^2^	<0.0001	a	4 × 10^3^	2	2 × 10^3^	7 × 10^1^	7 × 10^−6^	a	5 × 10^1^	2	3 × 10^1^	3 × 10^2^	9 × 10^−9^	a
PEG*PCL	1 × 10^−1^	1	1 × 10^−1^	3 × 10^2^	<0.0001	a	5 × 10^2^	1	5 × 10^2^	2 × 10^1^	3 × 10^−3^	a	1	1	1	1 × 10^1^	5 × 10^−3^	a
PEG*PE	3 × 10^−4^	1	3 × 10^−4^	8 × 10^−1^	4 × 10^−1^		1 × 10^−1^	1	1 × 10^−1^	6 × 10^−3^	9 × 10^−1^		1 × 10^−1^	1	1 × 10^−1^	1	3 × 10^−1^	
PCL*PE	3 × 10^−4^	1	3 × 10^−4^	8 × 10^−1^	4 × 10^−1^		2 × 10^−2^	1	2 × 10^−2^	8 × 10^−4^	1		9 × 10^−2^	1	9 × 10^−2^	9 × 10^−1^	4 × 10^−1^	
PEG*PCL*PE	7 × 10^−3^	1	7 × 10^−3^	2 × 10^1^	1 × 10^−3^	A	-	-	-	-	-		5	1	5	5 × 10^1^	6 × 10^−5^	a
Residual	3 × 10^−3^	9	3 × 10^−4^				2 × 10^2^	8	3 × 10^1^				9 × 10^−1^	9	1 × 10^−1^			
Lack of Fit	4 × 10^−4^	2	2 × 10^−4^	6 × 10^−1^	6 × 10^−1^	b	6	1	6	2 × 10^−1^	7 × 10^−1^	b	3 × 10^−2^	2	1 × 10^−2^	1 × 10^−1^	9 × 10^−1^	b
Pure Error	2 × 10^−3^	7	3 × 10^−4^				2 × 10^2^	7	3 × 10^1^				9 × 10^−1^	7	1 × 10^−1^			
Total	6 × 10^−1^	15					5 × 10^3^	13					6 × 10^1^	15				

a: Significant at the 95% level; b: Not significant at the 95% level; DF: Degrees of freedom; SS: Sum of squares; MS: Mean square; F: Ratio.

**Table 3:** ANOVA test of model regression for thermal stability, water absorption kinetics and hydrolytic degradation kinetics.

Source	Thermal Stability						Water Absorption Kinetics						Hydrolytic Degradation Kinetics					
	SS	DF	MS	F	*p*-Value		SS	DF	MS	F	*p*-Value		SS	DF	MS	F	*p*-Value	
Model	6 × 10^−1^	2	3 × 10^−1^	9 × 10^1^	4 × 10^−8^	a	2 × 10^8^	6	3 × 10^7^	2 × 10^3^	5 × 10^−12^	a	3 × 10^7^	6	6 × 10^6^	3 × 10^2^	6 × 10^−10^	a
Linear Mixture	6 × 10^−1^	2	3 × 10^−1^	9 × 10^1^	4 × 10^−8^	a	1 × 10^8^	2	5 × 10^7^	3 × 10^3^	2 × 10^−12^	a	3 × 10^7^	2	2 × 10^7^	9 × 10^2^	5 × 10^−11^	a
PEG*PCL	-	-	-	-	-		6 × 10^6^	1	6 × 10^6^	4 × 10^2^	4 × 10^−8^	a	3 × 10^6^	1	3 × 10^6^	1 × 10^2^	8 × 10^−7^	a
PEG*PE	-	-	-	-	-		1 × 10^5^	1	1 × 10^5^	8	2 × 10^−2^		4 × 10^4^	1	4 × 10^4^	2	2 × 10^−1^	
PCL*PE	-	-	-	-	-		2 × 10^4^	1	2 × 10^4^	1	3 × 10^−1^		3 × 10^4^	1	3 × 10^4^	2	2 × 10^−1^	
PEG*PCL*PE	-	-	-	-	-		1x 10^7^	1	1 × 10^7^	9 × 10^2^	2 × 10^−9^		3 × 10^6^	1	3 × 10^6^	1 × 10^2^	9 × 10^−7^	a
Residual	4 × 10^−2^	12	3 × 10^−3^				1 × 10^5^	8	1 × 10^4^				2 × 10^5^	9	2 × 10^4^			
Lack of Fit	2 × 10^−2^	6	4 × 10^−3^	1	5 × 10^−1^	b	4 × 10^4^	2	2 × 10^4^	1	3 × 10^−1^	b	3 × 10^4^	2	1 × 10^4^	8 × 10^−1^	5 × 10^−1^	b
Pure Error	2 × 10^−2^	6	3 × 10^−3^				8 × 10^4^	6	1 × 10^4^				1 × 10^5^	7	2 × 10^4^			
Total	7 × 10^−1^	14					2 × 10^8^	14					3 × 10^7^	15				

a: Significant at the 95% level; b: Not significant at the 95% level; DF: Degrees of freedom; SS: Sum of squares; MS: Mean square; F: Ratio.

**Table 4:** ANOVA of model regression of tensile strength, modulus and hardness.

Source	Tensile Strength						Modulus						Hardness					
	SS	DF	MS	F	*p*-Value		SS	DF	MS	F	*p*-Value		SS	DF	MS	F	*p*-Value	
Model	6	6	9 × 10^−1^	4 × 10^1^	4 × 10^−6^	a	5.4 × 10^−3^	6	9 × 10^−4^	2.9 × 10^2^	<0.0001	a	2 × 10^3^	6	4 × 10^2^	5 × 10^1^	2 × 10^−6^	a
Linear Mixture	4	2	2	9 × 10^1^	1 × 10^−6^	a	4 × 10^−3^	2	2 × 10^−3^	6.4 × 10^2^	<0.0001	a	8 × 10^2^	2	4 × 10^2^	6 × 10^1^	8 × 10^−6^	a
PEG*PCL	5 × 10^−2^	1	5 × 10^−2^	2	2 × 10^−1^		1 × 10^−4^	1	1 × 10^−4^	3.6 × 10^1^	2 × 10^−4^	a	2 × 10^1^	1	2 × 10^1^	3	1 × 10^−1^	
PEG*PE	2 × 10^−2^	1	2 × 10^−2^	1	3 × 10^−1^		1 × 10^−3^	1	1 × 10^−3^	4.3 × 10^−1^	5. × 10^−1^		4	1	4	6 × 10^−1^	5 × 10^−1^	
PCL*PE	2 × 10^−2^	1	2 × 10^−2^	7 × 10^−1^	4 × 10^−1^		6 × 10^−5^	1	6 × 10^−5^	1.8 × 10^−2^	8.9 × 10^−1^		4	1	4	6 × 10^−1^	5 × 10^−1^	
PEG*PCL*PE	5 × 10^−1^	1	5 × 10^−1^	2 × 10^1^	1 × 10^−3^	b	5 × 10^−4^	1	5 × 10^−4^	1.4 × 10^2^	<0.0001	a	1 × 10^3^	1	1 × 10^3^	1 × 10^2^	1 × 10^−6^	a
Residual	2 × 10^−1^	9	2 × 10^−2^				0.0000	9	3 × 10^−3^				7 × 10^1^	9	7			
Lack of Fit	5 × 10^−2^	2	3 × 10^−2^	1	4 × 10^−1^	b	3 × 10^−3^	2	2 × 10^−3^	4.6 × 10^−1^	6.5 × 10^−1^	b	2 × 10^1^	2	1 × 10^1^	2	2 × 10^−1^	b
Pure Error	1 × 10^−1^	7	2 × 10^−2^				0.0000	7	4 × 10^−3^				4 × 10^1^	7	6			
Total	6	15					5.4 × 10^−3^	15					2 × 10^3^	15				

a: Significant at the 95% level; b: Not significant at the 95% level; DF: Degrees of freedom; SS: Sum of squares; MS: Mean square; F: Ratio.

**Table 5:** Onset temperatures of PUs synthetized.

Sample	Onset Temperature (°C)
S1	273.21
S2	276.84
S3	264.15
S4	263.92
S5	274.08
S6	262.17
S7	254.05
S8	268.65
S9	260.56
S10	253.74
S11	258.31
S12	264.82
S13	262.20
S14	261.75
S15	225.60
S16	263.08

**Table 6:** Variation ranges in experimental design for polyurethanes synthesis.

Blend Component	Lower Limit *	Upper Limit *
PEG	0.000	0.900
PCL	0.000	0.900
PE	0.050	0.100

* Mass fraction.

**Table 7:** Description of mixture design for the study of polyol blend composition.

Polyol Blend	Type	Sample Name	PEG *	PCL *	PE *	Note
Binary	Vertex	S1	0.000	0.900	0.100	
Binary	Vertex	S2	0.000	0.900	0.100	Repetition of S1
All components	Edge centroid	S3	0.025	0.900	0.075	
All components	Vertex	S4	0.050	0.900	0.050	
All components	Vertex	S5	0.050	0.900	0.050	Repetition of S4
All components	Edge centroid	S6	0.450	0.450	0.100	
All components	Edge centroid	S7	0.450	0.450	0.100	Repetition of S6
All components	Edge centroid	S8	0.450	0.450	0.100	Repetition of S6
All components	Overall centroid	S9	0.463	0.463	0.075	
All components	Edge centroid	S10	0.475	0.475	0.050	
All components	Edge centroid	S11	0.475	0.475	0.050	Repetition of S10
Binary	Vertex	S12	0.900	0.000	0.100	
Binary	Vertex	S13	0.900	0.000	0.100	Repetition of S12
Binary	Vertex	S14	0.900	0.000	0.100	Repetition of S12
All components	Edge centroid	S15	0.900	0.025	0.075	
All components	Vertex	S16	0.900	0.050	0.050	
Check Point		C1	0.571	0.33	0.099	
Check Point		C2	0.815	0.091	0.094	
Check Point		C3	0.796	0.107	0.097	

* Mass fraction.

## Supplementary Materials

The supplementary materials are available online.
