# Supplementary material for: Surface Response Methodology-Based Mixture Design to Study the Influence of Polyol Blend Composition on Polyurethanes’ Properties
Source: Molecules. 2018 Aug 3;23(8):1942. doi: 10.3390/molecules23081942 (PMC6222905; doi:10.3390/molecules23081942)
Supplement: Supplementary file 1 [file molecules-23-01942-s001.zip › molecules-329206-supplementary.docx]

Table S1: Raw data of polyurethane properties used for the estimation of regression models and parameters

| Sample | Water absorption (%) | Hydrolytic degradation (%) | Contact angle (Degree) | Ultimate strengh (MPa) | Elongation at break (%) | Hardness (Share A) | Modulus (MPa) | Area under the curve – hydrolytic degradation (h) | Area under the curve – Water absorption (h) | Area under the curve – DTGs (1/°C) |
| --- | --- | --- | --- | --- | --- | --- | --- | --- | --- | --- |
| S1 | 2,27605 | 1,51396 | 102,764 | 7,02441 | 148,489 | 88,25 | 0,0613834 | 133,053 | 254,445 | 5,645 |
| S2 | 2,52504 | 0,87772 | 105,755 | 7,96776 | 108,002 | 88,125 | 0,0649609 | 156,138 | 234,87 | 5,52 |
| S3 | 3,14466 | 1,18102 | 104,027 | 2,97568 | 89,6833 | 81,25 | 0,0399291 | 152,558 | 224,186 | 5,58 |
| S4 | 3,9117 | 0,233608 | 95,2303 | 1,87664 | 139,256 | 65,625 | 0,0205689 | 153,553 | 403,21 | 5,625 |
| S5 | 3,48757 | 0,64387 | 103,451 | 1,6281 | 136,293 | 64,625 | 0,0171809 | 130,207 | 267,45 | 5,65 |
| S6 | 32,4151 | 10,1392 | 66,4663 | 1,83004 | 105,551 | 72,375 | 0,0272378 | 1107,7 | 3614,7 | 5,31 |
| S7 | 33,7842 | 6,70871 | 72,806 | 2,97451 | 133,728 | 78,75 | 0,0249384 | 734,864 | 3824,46 |  |
| S8 | 30,7145 | 6,04405 | 71,9217 | 2,92448 | 114,474 | 75 | 0,0264773 | 848,1 | 3531,44 | 5,4 |
| S9 | 26,9397 | 14,8045 | 60,6796 | 2,86414 | 129,357 | 80,625 | 0,0266628 | 1839,8 | 3019,94 | 5,34 |
| S10 | 24,2522 | 19,3777 | 48,701 | 2,67 |  | 89,5 | 0,030327 | 2246,66 | 3280,79 | 5,42 |
| S11 | 33,3416 | 15,5442 | 60,2976 | 2,87547 | 162,313 | 88,75 | 0,0274975 | 2046,15 |  | 5,43 |
| S12 | 23,949 | 43,1348 | 69,8157 | 0,469674 |  | 70,625 | 0,00204419 | 4189,18 | 3700,62 | 5,1 |
| S13 | 34,5516 | 37,1792 | 69,2569 | 0,918028 | 176,601 | 66,875 | 0,00525863 | 3932,25 | 3510,65 | 5,16 |
| S14 | 36,7869 | 32,5548 | 59,4589 | 0,711823 | 164,622 | 63,875 | 0,00473538 | 3948,15 | 3502,61 | 5,04 |
| S15 | 72,7102 | 27,5029 |  | 0,653554 |  | 58,875 | 0,00519083 | 3469,32 | 7899,53 | 5,16 |
| S16 | 109,208 | 18,1811 |  | 0,656179 |  | 45,875 | 0,00336487 | 2750,8 | 12478,3 | 5,03 |

Empty spaces correspond to values deleted by the DFFITS tests

Table S2: Time depend data for water absorption, in % of water swelling, used to calculate the area under the curves by the trapezoid method

| **Sample** | **Time (h)** | | | | | | |
| --- | --- | --- | --- | --- | --- | --- | --- |
|  | **9** | **25** | **29** | **31** | **53** | **69** | **120** |
| S1 | 2.89 | 2.51 | 1.39 | 2.28 | 0.40 | 1.43 | 4.16 |
| S2 | 2.74 | 2.19 | 2.05 | 2.33 | 1.77 | 1.36 | 2.57 |
| S3 | 2.11 | 2.36 | 2.37 | 1.99 | 1.33 | 1.91 | 2.12 |
| S4 | 3.72 | 3.23 | 3.70 | 3.07 | 1.55 | 3.34 | 5.30 |
| S5 | 3.10 | 2.36 | 2.07 | 2.45 | 2.36 | 2.17 | 2.04 |
| S6 | 33.00 | 32.50 | 30.43 | 36.10 | 29.79 | 33.01 | 26.70 |
| S7 | 36.38 | 35.10 | 34.80 | 35.31 | 33.25 | 31.48 | 31.54 |
| S8 | 35.74 | 32.95 | 33.18 | 30.95 | 30.59 | 30.00 | 27.37 |
| S9 | 35.02 | 27.33 | 27.65 | 27.80 | 26.85 | 20.98 | 26.64 |
| S10 | 28.83 | 51.57 | 23.98 | 26.75 | 24.67 | 26.95 | 25.11 |
| S11 | 33.91 | 29.88 | 32.38 | 29.88 | 35.07 | 29.10 | 38.19 |
| S12 | 37.91 | 33.52 | 32.70 | 33.03 | 30.03 | 30.99 | 30.91 |
| S13 | 43.18 | 33.20 | 31.91 | 33.07 | 29.87 | 26.08 | 27.65 |
| S14 | 42.25 | 31.40 | 27.38 | 30.53 | 30.64 | 28.70 | 26.21 |
| S15 | 79.32 | 69.53 | 72.06 | 67.50 | 67.71 | 67.48 | 64.29 |
| S16 | 125.92 | 109.26 | 107.99 | 107.36 | 106.18 | 105.73 | 103.54 |

Table S3: Time depend data for hydrolytic degradation, in % weight loss, used to calculate the area under the curves by the trapezoid method

| **Sample** | **Time (h)** | | | | |
| --- | --- | --- | --- | --- | --- |
|  | **9** | **29** | **55** | **71** | **120** |
| S1 | 1.36 | 1.05 | 1.99 | 0.42 | 1.39 |
| S2 | 1.32 | 0.98 | 1.24 | 1.72 | 1.33 |
| S3 | 2.36 | 0.49 | 1.59 | 1.30 | 1.27 |
| S4 | 1.50 | 0.89 | 1.13 | 1.93 | 1.01 |
| S5 | 1.67 | 1.09 | 1.28 | 0.93 | 0.98 |
| S6 | 7.83 | 11.96 | 10.42 | 7.20 | 10.88 |
| S7 | 4.84 | 6.42 | 6.49 | 6.75 | 6.59 |
| S8 | 4.87 | 6.38 | 5.64 | 8.61 | 9.48 |
| S9 | 13.21 | 15.36 | 16.21 | 18.19 | 14.83 |
| S10 | 18.25 | 19.51 | 19.57 | 20.38 | 18.77 |
| S11 | 17.08 | 17.54 | 17.34 | 18.37 | 17.71 |
| S12 | 35.36 | 36.70 | 36.53 | 36.55 | 35.81 |
| S13 | 30.76 | 34.41 | 34.27 | 36.20 | 32.59 |
| S14 | 30.49 | 36.03 | 33.98 | 35.03 | 33.68 |
| S15 | 28.25 | 29.93 | 30.33 | 30.24 | 30.67 |
| S16 | 20.22 | 24.05 | 24.88 | 24.41 | 24.04 |
